# Reduction of *Kcnt1* is therapeutic in mouse models of *SCN1A* and *SCN8A* epilepsy

**DOI:** 10.3389/fnins.2023.1282201

**Published:** 2023-10-13

**Authors:** Sophie F. Hill, Paymaan Jafar-Nejad, Frank Rigo, Miriam H. Meisler

**Affiliations:** ^1^Neuroscience Graduate Program, University of Michigan, Ann Arbor, MI, United States; ^2^Department of Human Genetics, University of Michigan, Ann Arbor, MI, United States; ^3^Ionis Pharmaceuticals, Carlsbad, CA, United States; ^4^Department of Neurology, University of Michigan, Ann Arbor, MI, United States

**Keywords:** *Scn8a*, *Scn1a*, *Kcnt1*, epilepsy, ASO, sodium channel, potassium channel

## Abstract

Developmental and epileptic encephalopathies (DEEs) are severe seizure disorders with inadequate treatment options. Gain- or loss-of-function mutations of neuronal ion channel genes, including potassium channels and voltage-gated sodium channels, are common causes of DEE. We previously demonstrated that reduced expression of the sodium channel gene *Scn8a* is therapeutic in mouse models of sodium and potassium channel mutations. In the current study, we tested whether reducing expression of the potassium channel gene *Kcnt1* would be therapeutic in mice with mutation of the sodium channel genes *Scn1a* or *Scn8a*. A *Kcnt1* antisense oligonucleotide (ASO) prolonged survival of both *Scn1a* and *Scn8a* mutant mice, suggesting a modulatory effect for KCNT1 on the balance between excitation and inhibition. The cation channel blocker quinidine was not effective in prolonging survival of the *Scn8a* mutant. Our results implicate *KCNT1* as a therapeutic target for treatment of *SCN1A* and *SCN8A* epilepsy.

## 1. Introduction

Developmental and epileptic encephalopathies (DEEs) are among the most severe epileptic disorders. The typical disease course begins with onset of seizures during the first year of life, followed by developmental delay, movement disorders, intellectual disability, sleep disturbances, and feeding difficulties (Scheffer and Nabbout, 2019; Meisler et al., 2021; Johannesen et al., 2022). Seizures are often resistant to treatment with current antiepileptic drugs (Scheffer and Nabbout, 2019; Meisler et al., 2021; Johannesen et al., 2022).

Many DEEs result from mutations in sodium and potassium channel genes (Lindy et al., 2018; Symonds et al., 2019). Based on their roles in the neuronal action potential, excessive sodium current or insufficient potassium current would be predicted to cause hyperexcitability and epilepsy. In agreement with expectation, many missense mutations in the voltage-gated sodium channel gene *SCN8A* result in excessive sodium current (“gain-of-function”, or GOF, mutations) and *SCN8A*-DEE (Veeramah et al., 2012; Meisler et al., 2021; Johannesen et al., 2022). Experimental expression of an *SCN8A* GOF mutation in excitatory neurons is sufficient to cause seizures and premature death, while expression limited to inhibitory neurons does not (Bunton-Stasyshyn et al., 2019).

Loss-of-function (LOF) mutations of the sodium channel gene *SCN1A* and GOF mutations of the potassium channel gene *KCNT1* can also cause epilepsy (Barcia et al., 2012; Scheffer and Nabbout, 2019; Gribkoff and Winquist, 2023). *SCN1A* haploinsufficiency reduces the excitability of inhibitory neurons, altering excitation/inhibition balance (Cheah et al., 2012; Tai et al., 2014; Favero et al., 2018). The epileptogenic mechanism of *KCNT1* GOF mutations is not well established, but a similar disinhibitory mechanism may be involved (Shore et al., 2020; Gertler et al., 2022; Wu et al., 2023).

KCNT1 is a sodium-activated potassium channel (also known as Slo2.2, K_Na_1.1, or Slack) with widespread expression in the central nervous system (Rizzi et al., 2016). KCNT1 regulates afterhyperpolarization amplitude and action potential threshold (Martinez-Espinosa et al., 2015; Quraishi et al., 2019; Shore et al., 2020; Gertler et al., 2022; Wu et al., 2023). *KCNT1* GOF mutations enhance bursting behavior in excitatory neurons and reduce action potential firing in inhibitory neurons (Quraishi et al., 2019; Shore et al., 2020; Gertler et al., 2022; Wu et al., 2023).

In the mouse, homozygous knock-in of *KCNT1* GOF mutations results in spontaneous seizures, reduced threshold for seizure induction, behavioral abnormalities, and premature lethality (Quraishi et al., 2020; Shore et al., 2020; Burbano et al., 2022; Gertler et al., 2022). Burbano et al. (2022) described an antisense oligonucleotide (ASO) that reduces expression of *Kcnt1*. Administration of the ASO to a homozygous *Kcnt1* GOF mouse prolonged survival, reduced seizure frequency, and corrected behavioral abnormalities. Conversely, homozygous loss of *Kcnt1* also improves survival after electrically induced seizrues (Quraishi et al., 2020).

Reducing expression of *Scn8a* prolongs survival of epileptic mice with mutations in the potassium channel genes *Kcna1* and *Kcnq2* (Hill et al., 2022). Here, we asked whether modulating expression of a potassium channel can improve the phenotype of sodium channel mutants. Administration of the *Kcnt1* ASO (Burbano et al., 2022) on postnatal day 2 doubled the lifespan of *Scn8a* mutant mice and extended survival of *Scn1a* haploinsufficient mice. Our results suggest a new therapeutic intervention for DEEs caused by mutations of *SCN1A* and *SCN8A*.

## 2. Methods

### 2.1. Mice

The *Scn8a^cond^* allele, abbreviated W, contains two tandem copies of exon 26, the final coding exon of *Scn8a* (Bunton-Stasyshyn et al., 2019). The upstream copy, designated 26a, is a floxed exon that encodes the wildtype channel. Deletion of exon 26a by Cre results in expression of exon 26b encoding the variant p.R1872W. This variant has been identified in multiple individuals with *SCN8A* epilepsy (Bunton-Stasyshyn et al., 2019; Johannesen et al., 2022). *Scn8a^cond/cond^* male mice were crossed with *EIIa-Cre*/+ female mice (JAX 003724) to generate *Scn8a^cond/+^,EIIa-Cre* double heterozygous mice expressing the R1872W variant (designated W/+ mice). Both the *Scn8a^cond^* allele and the *EIIa-Cre* transgene were maintained on a C57Bl/6 J genetic background.

*Scn1a^+/−^* mice with deletion of exon 1 were maintained on the protective 129S6/SvEvTac strain background and activated in (C57BL/6 J X 129S6/SvEvTac) F1 mice (Miller et al., 2014). Both male and female mice were used for all experiments. Experiments were approved by the Committee on the Use and Care of Animals at the University of Michigan.

### 2.2. ASOs

ASOs were synthesized by Ionis Pharmaceuticals as described (Swayze et al., 2007). Both the non-targeting control and *Kcnt1* ASOs are 20-bp gap-mers with 5 2’-*O*-methoxyethyl modifications on the first and last 5 bases and phosphorothioate modifications on all 20 bases. The *Kcnt1* ASO (5’ GCT TCA TGC CAC TTT CCA GA 3′) is complementary to the 3’ UTR of mouse *Kcnt1* and was previously described (Burbano et al., 2022). The non-targeting control ASO (5’ CCT ATA GGA CTA TTC AGG AA 3′) is well-tolerated and is not complementary to any transcript encoded by the mouse genome (Swayze et al., 2007). Animals treated with control ASO received a 30 μg dose.

### 2.3. Intracerebroventricular (ICV) injections

At postnatal day 2 (P2), mice were cryo-anesthetized for 3 min. ASO was diluted in PBS (2 μL injection volume) and manually injected into the left ventricle as described (Lenk et al., 2020). Animals were allowed to recover for 10 min at 37°C before being returned to the home cage.

### 2.4. qRT-PCR

Brain and spinal cord from 3-week-old mice treated with control or *Kcnt1* ASO were homogenized in TRIzol (Invitrogen Cat. #15596026, Waltham, MA). RNA was extracted using the Direct-zol RNA Mini Prep kit from Zymo Research (Irvina, CA). cDNA was synthesized with the LunaScript kit from New England Biolabs (Ipwsich, MA). *Scn8a* (Mm00488110_m1), *Kcnt1* (Mm01330661_g1), and *Tbp* (Mm01277042_m1) transcripts were quantified using TaqMan gene expression assays (Applied Biosystems, Foster City, CA).

### 2.5. Quinidine administration

Quinidine (Sigma Aldrich, St. Louis, MO) was diluted in phosphate-buffered saline (50 or 100 mg/kg) and administered by daily intraperitoneal injection beginning at P10, the youngest age at which daily intraperitoneal injections were feasible.

## 3. Results

### 3.1. Characterization of the *Kcnt1* ASO

**Figure 1:**
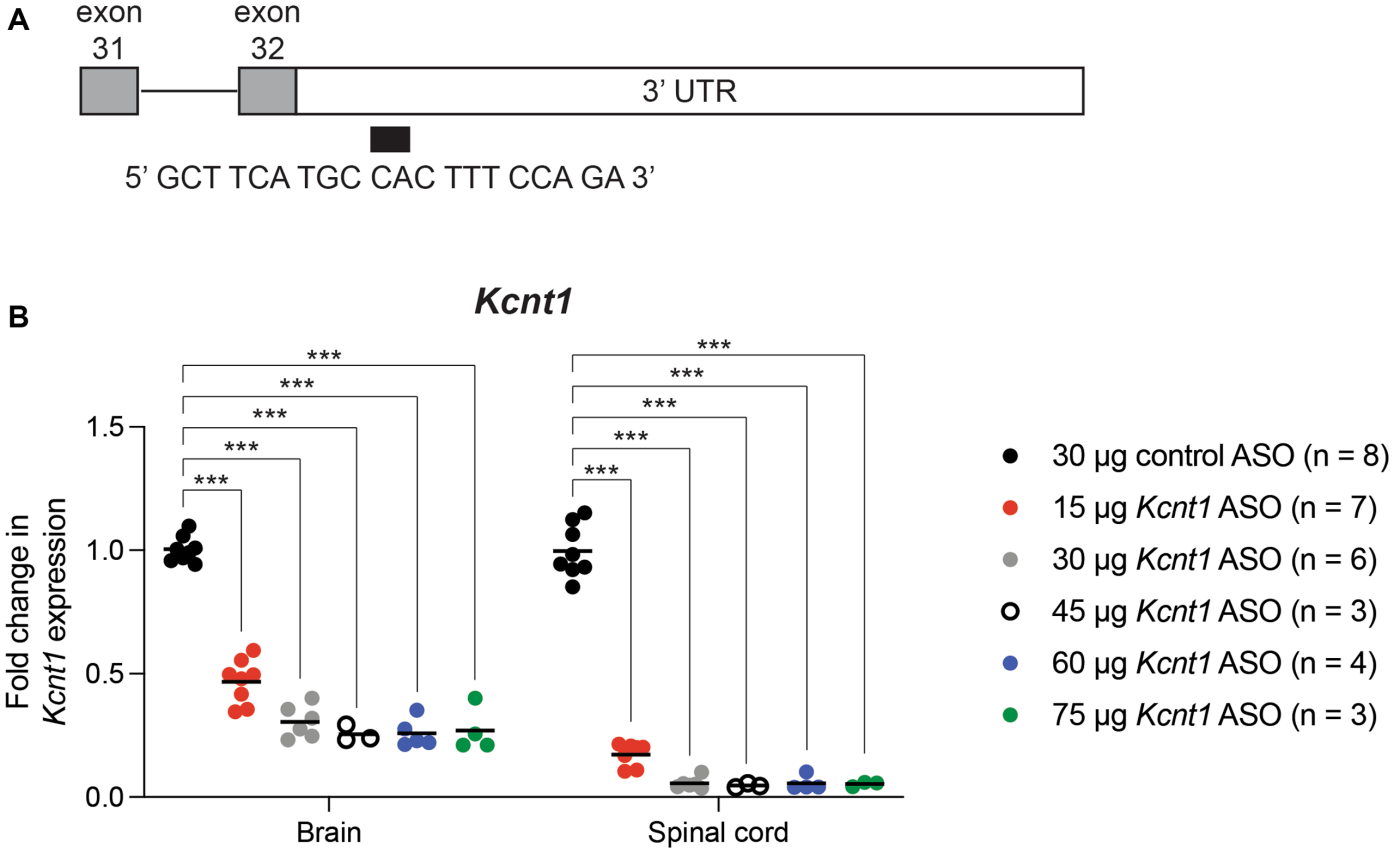
*Kcnt1* ASO reduces *Kcnt1* transcript abundance. **(A)** The *Kcnt1* ASO targets the proximal 3’UTR of the *Kcnt1* transcript. **(B)** Expression of *Kcnt1* in brain and spinal cord from P21 wildtype mice treated with *Kcnt1* ASO on P2, measured by qRT-PCR (*** = *p* < 0.0001, Sidak’s multiple comparisons test).

We used an ASO to reduce expression of mouse *Kcnt1*. The 20 base-pair “gap-mer” ASO targets the 3’ UTR of the mouse *Kcnt1* gene (Figure 1A) and recruits RNaseH1 to degrade the transcript (Burbano et al., 2022). We first administered the ASO to wild-type animals by ICV injection at P2. Three weeks later, we measured gene expression in brain and spinal cord by qRT-PCR (Figure 1B; Supplementary Table S1). *Kcnt1* expression was reduced in both brain and spinal cord (two-way ANOVA, *p* < 0.0001). For example, adminsitration of 45 μg *Kcnt1* ASO reduced *Kcnt1* expression in brain to 0.25 ± 0.04 of control (mean ± SD, *n* = 3) (Supplementary Table S1). Reduction of *Kcnt1* transcript reduces KCNT1 protein expression (Burbano et al., 2022). Expression of *Scn8a* was unaffected by the *Kcnt1* ASO (Supplementary Figure S1). No changes in *Kcnt1* expression were detected in previous studies of *Scn1a* and *Scn8a* mutant mice (Sprissler et al., 2017; Hawkins et al., 2019; Valassina et al., 2022).

### 3.2. ASO-mediated reduction of *Kcnt1* extends the lifespan of an *SCN8A* DEE mouse

**Figure 2:**
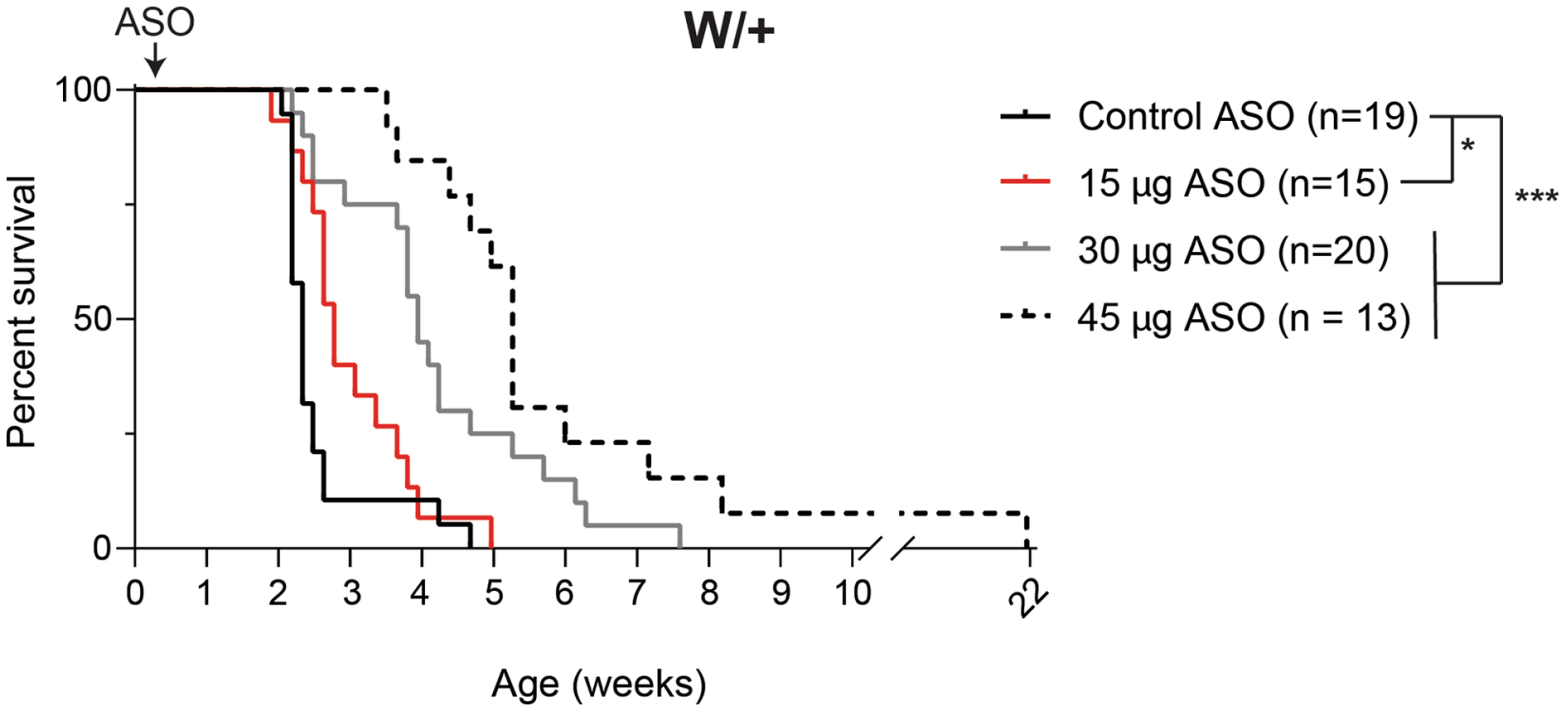
*Kcnt1* ASO prolongs survival of *Scn8a* mutant mice. Survival of *Scn8a^cond/+^,EIIa-Cre* (W/+) mice treated with 15–45 μg *Kcnt1* ASO at P2 compared with a cohort of contemporaneous control mice treated with control ASO [Control data was previously published in Lenk et al. (2020)]. Asterisks indicate significance of Mantel-Cox log-rank tests: * = *p* < 0.05, *** = *p* < 0.0001.

We previously generated a mouse with Cre-dependent expression of the patient mutation p.R1872W (Bunton-Stasyshyn et al., 2019). Expression of this mutation by crossing with the ubiquitously expressed *EIIa-Cre* results in a single, lethal seizure at P14 (Bunton-Stasyshyn et al., 2019). We treated *Scn8a^cond/+^,EIIa-Cre* (W/+) animals with 15–75 μg *Kcnt1* ASO by ICV injection at P2. Mice treated with the control ASO exhibited median survival of 16 days (Figure 2). Mice treated with 15 μg *Kcnt1* ASO lived three days longer (median survival = 19 days, *p* = 0.0493, Mantel-Cox log-rank test). Treatment with 30 μg *Kcnt1* ASO extended median survival to 27 days (*p* < 0.0001, Mantel- Cox log-rank test). Mice treated with 45 μg, the optimal dose, exhibited median survival of 36 days, more than double the lifespan of control ASO-treated mice (*p* < 0.0001, Mantel-Cox log-rank test, Figure 2). Treatment with 60 or 75 μg *Kcnt1* ASO did not further reduce *Kcnt1* expression (Figure 1B) or further extend survival (Supplementary Figure S2).

### 3.3. Quinidine does not extend survival in the *SCN8A* DEE mouse

Quinidine is a nonspecific cation channel blocker used to treat cardiac arrhythmia. *In vitro*, quinidine blocks KCNT1 channel activity, suggesting that it could be a precision therapy for patients with gain-of-function *KCNT1* mutations (Mori et al., 1998; Milligan et al., 2014). *In vivo*, quinidine has mixed efficacy in *KCNT1* epilepsy patients (Mikati et al., 2015; Numis et al., 2018; Fitzgerald et al., 2019; Cole et al., 2021).

**Figure 3:**
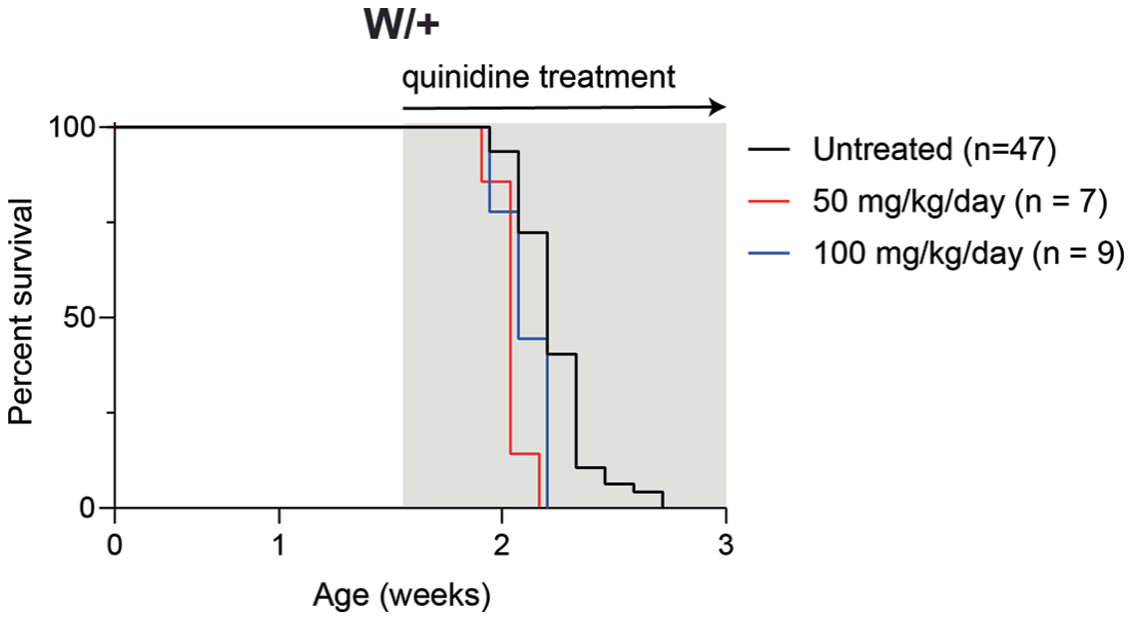
Quinidine does not prolong survival of *Scn8a* mutant mice. Survival of *Scn8a^cond/+^,EIIa-Cre* (W/+) mice daily treated with 50 or 100 mg/kg quinidine compared to untreated mice. Grey shading indicates treatment period.

To determine whether inhibition of KCNT1 channels by quinidine would be therapeutic in *Scn8a* mutant mice, we administered 50 or 100 mg/kg quinidine by daily intraperitoneal injection beginning at P10 (Figure 3). Untreated mice exhibited median survival of 15 days (*n* = 47). Treatment with 50 or 100 mg/kg quinidine did not extend the lifespan of the *SCN8A*-DEE mice (median survival = 14 days; *n* = 7 & 9, respectively; Figure 3).

### 3.4. ASO-mediated reduction of *Kcnt1* extends the lifespan of a mouse model of *SCN1A* haploinsufficiency

**Figure 4:**
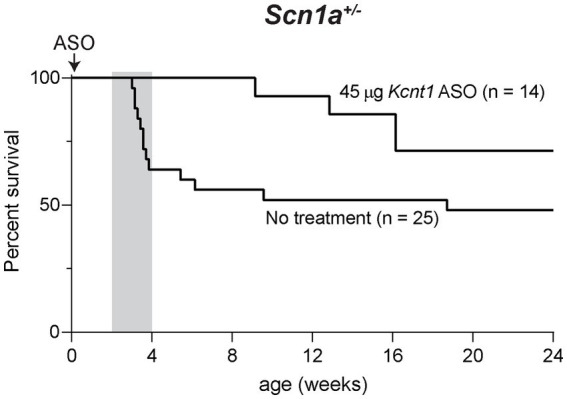
*Kcnt1* ASO prolongs survival of *Scn1a^+/−^* mice. Survival of *Scn1a^+/−^* mice treated with 45 μg *Kcnt1* ASO at P2 compared to untreated mice (*p* = 0.0513, Mantel-Cox log-rank test). Grey shading indicates the critical period in the development of Dravet Syndrome.

We also tested the effect of the *Kcnt1* ASO in *Scn1a^+/−^* mice, a model of Dravet Syndrome. Consistent with previous studies (Miller et al., 2014; Favero et al., 2018), approximately 1/3 of untreated *Scn1a^+/−^* mice died between 3 and 4 weeks of age, and during the remaining 6-month monitoring period, there were several sporadic deaths (Figure 4). We administered 45 μg *Kcnt1* ASO to *Scn1a^+/−^* mice at P2. None of the treated mice died in the first 4 weeks, indicating that reduced *Kcnt1* expression during this critical period is sufficient to prevent death (*p* = 0.0513, Mantel-Cox log-rank test, Figure 4). There were four deaths during the 6-month monitoring period, all after 9 weeks of age (Figure 4). Since quinidine was not effective in the *Scn8a* mutant mice, we did not treat the *Scn1a* mutant mice.

## 4. Discussion

Developmental and epileptic encephalopathies are frequently caused by pathological variants of ion channel genes. Here, we showed that reduction of *Kcnt1* expression is protective in mouse models of *Scn1a* and *Scn8a* epilepsy. Our findings suggest that patients with mutations of *SCN1A* and *SCN8A* could benefit from treatment with a *KCNT1* ASO or KCNT1-specific channel blocker.

A previous study demonstrated that the *Kcnt1* ASO improved the survival, seizure, and behavioral phenotypes of *Kcnt1* GOF mice (Burbano et al., 2022). Interestingly, the *Kcnt1* ASO was therapeutic at lower doses in *Kcnt1* mutant mice than in the *Scn8a* mutant studied here. In neonatal *Kcnt1* mutant mice, 3.4 μg extended the median survival by more than 100 days (Burbano et al., 2022). In contrast, doses of 15–45 μg added only 20 days to survival of the *Scn8a* mutant mice. These observations suggest that the effect in the *Scn8a* mutant may be indirect. For example, gain-of-function of *Kcnt1* reduces excitability of parvalbumin interneurons (Gertler et al., 2022). Reduced expression of *Kcnt1* may enhance excitability of parvalbumin interneurons and thereby reduce seizure susceptibility. Consistent with this hypothesis, homozygous knockout of *Kcnt1* reduces the lethality of electrically-induced seizures by more than half (Quraishi et al., 2020). Further investigation may identify other types of epilepsy that respond to reduction of *KCNT1*.

We previously demonstrated that reducing *Scn8a* expression is therapeutic in *Scn1a^+/−^* mice (Lenk et al., 2020) and in mice with epilepsy caused by loss of the potassium channel genes *Kcna1* and *Kcnq2* (Hill et al., 2022). P2 administration of the *Scn8a* ASO completely rescued the *Scn1a^+/−^* mice (Lenk et al., 2020). In contrast, 4/14 of the *Scn1a^+/−^* mice treated with the *Kcnt1* ASO died between two and six months of age. The deaths after 2 months may result from turnover of the *Kcnt1* ASO; alternatively, reduced *Kcnt1* may be effective only in the interval between 3–4 weeks. The long-term effectiveness of the *Scn8a* ASO in *Scn1a* mutant mice is interesting, since the effect on *Scn8a* expression persists for only 6 weeks (Lenk et al., 2020). Viral overexpression of the *Kcna1* channel is protective against seizures induced by tetanus neurotoxin or pentylenetetrazole (Snowball et al., 2019; Qiu et al., 2022). Taken together, these observations suggest that modulation of ion channel expression to compensate for epileptogenic mutations is a promising therapeutic strategy.

Among the ion channel genes that could be targeted to treat channelopathies, *KCNT1* is an attractive choice because reduced expression is well tolerated. Heterozygous loss-of-function mutations of *KCNT1* are present in the general population and not associated with disease (Lek et al., 2016; Karczewski et al., 2020). *Kcnt1^−/−^* mice are healthy and fertile, with minor abnormalities such as impaired reversal learning and slightly elevated pain sensitivity (Bausch et al., 2015; Lu et al., 2015; Martinez-Espinosa et al., 2015; Quraishi et al., 2020). In contrast, heterozygous loss of *Scn8a* is not present in the healthy population (probability of loss-of-function intolerance, pLI = 1) (Lek et al., 2016; Karczewski et al., 2020) and homozygous loss is lethal in the mouse (Burgess et al., 1995).

Quinidine has been proposed as a therapy for patients with *KCNT1* epilepsy because of the effectiveness of high doses for correction of GOF mutations *in vitro* (Milligan et al., 2014; Numis et al., 2018). The effects of quinidine are not specific to KCNT1 (Roden, 2014). Clinical application of quinidine in *KCNT1* epilepsy has mixed success. Some individuals achieved seizure freedom (Mikati et al., 2015; Fitzgerald et al., 2019), but most patients report no benefit or worsening seizures (Mikati et al., 2015; Numis et al., 2018; Cole et al., 2020). Quinidine concentration sufficiently high to block KCNT1 may be difficult to achieve *in vivo* without deleterious effects on other ion channels (Liu et al., 2022). We found that quinidine was not protective in *Scn8a* mutant mice. More specific KCNT1 channel blockers (Cole et al., 2020; Griffin et al., 2021) may be more effective for treatment of *KCNT1*, *SCN8A*, and *SCN1A* epilepsy.

## Data availability statement

The original contributions presented in the study are included in the article/Supplementary material, further inquiries can be directed to the corresponding author.

## Ethics statement

The animal study was approved by University of Michigan Institutional Animal Care & Use Committee. The study was conducted in accordance with the local legislation and institutional requirements.

## Author contributions

SH: Conceptualization, Data curation, Formal Analysis, Investigation, Methodology, Visualization, Writing – original draft, Writing – review & editing. PJ-N: Conceptualization, Resources, Writing – review & editing. FR: Conceptualization, Resources, Writing – review & editing. MM: Conceptualization, Funding acquisition, Supervision, Writing – review & editing.
