# Supplementary material for: Reduction of Kcnt1 is therapeutic in mouse models of SCN1A and SCN8A epilepsy
Source: Front Neurosci. 2023 Oct 13;17:1282201. doi: 10.3389/fnins.2023.1282201 (PMC10603267; doi:10.3389/fnins.2023.1282201)
Supplement: Supplementary file 1 [file Data_Sheet_1.docx]

Reduction of *Kcnt1* is therapeutic in mouse models of *SCN8A* and *SCN1A* epilepsy

Sophie Hill*, Paymaan Jafar-Nejad, Frank Rigo, and Miriam Meisler

*** Correspondence:** Sophie Hill: [sfhill@umich.edu](mailto:sfhill@umich.edu)

# Supplementary Figures and Tables

For more information on Supplementary Material and for details on the different file types accepted, please see [here](https://www.frontiersin.org/guidelines/author-guidelines#supplementary-material).

## Supplementary Figure 1

**Supplementary Figure 1.** ***Kcnt1* ASO does not affect *Scn8a* expression.** Expression of *Scn8a* in brain and spinal cord from P21 wildtype mice treated with *Kcnt1* ASO on P2, measured by qRT-PCR.

## Supplementary Figure 2


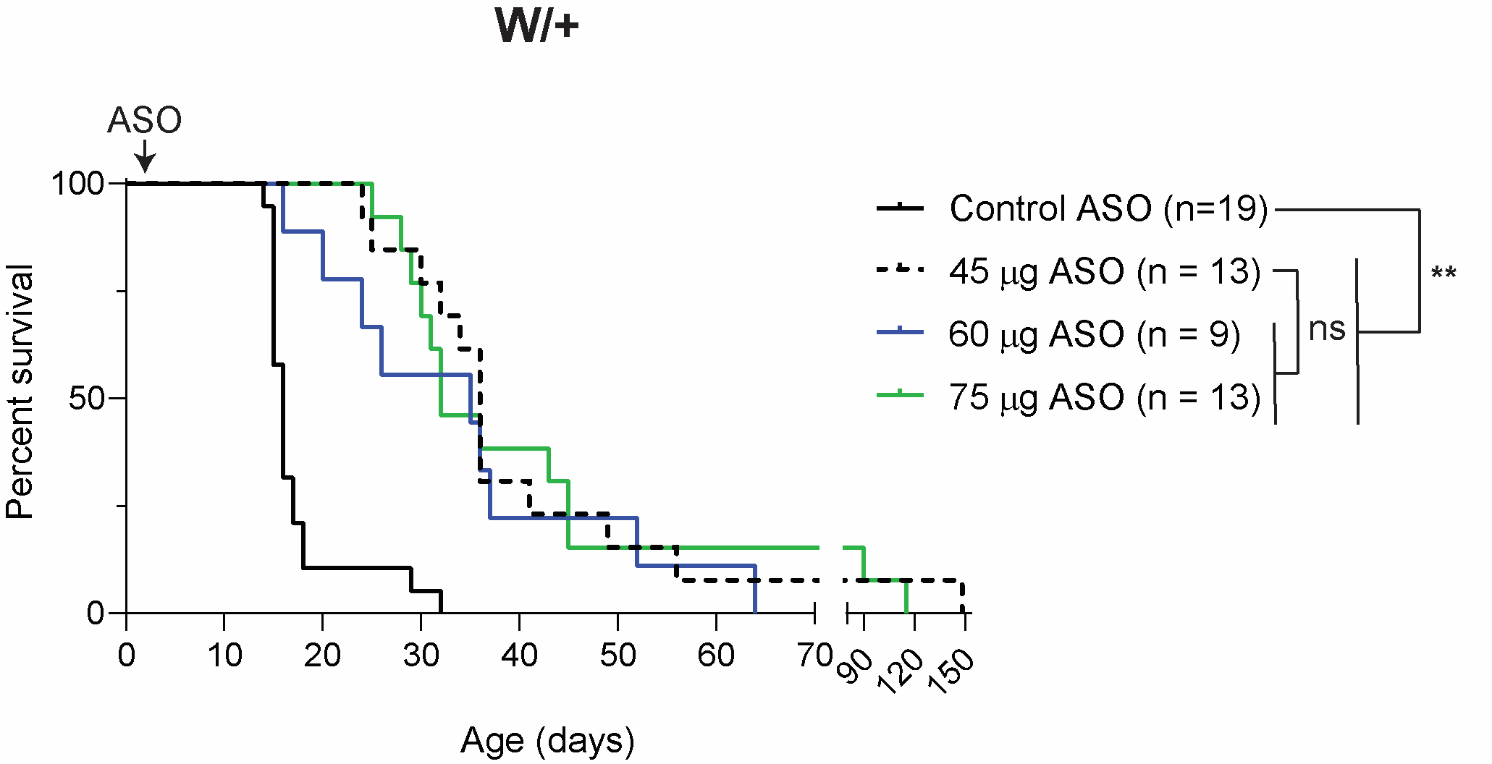


**Supplementary Figure 2. Higher doses of *Kcnt1* ASO (above 45** µg**) do not confer additional survival benefit in *Scn8a* mutant mice**. Survival of *Scn8a^cond/+^*,*EIIa-Cre* (W/+) mice treated at P2 with 45-75 µg *Kcnt1* ASO compared with previously-published mice treated with control ASO (Lenk *et al.*, 2020). Asterisks indicate significance of Mantel-Cox log-rank tests: ** = *p* < 0.005, ns = not significant.

## Supplementary Table 1

**Supplementary Table 1. *Kcnt1* ASO reduces *Kcnt1* transcript abundance.** Fold change of *Kcnt1* expression compared to mice treated with control ASO. Values are shown as mean ± standard deviation. The number of mice is shown in parentheses.

| ASO | Brain | Spinal Cord |
| --- | --- | --- |
| 30 μg control | 1.00 ± 0.05 (8) | 1.00 ± 0.11 (8) |
| 15 μg *Kcnt1* | 0.47 ± 0.09 (8) | 0.17 ± 0.05 (7) |
| 30 μg *Kcnt1* | 0.30 ± 0.07 (6) | 0.06 ± 0.02 (6) |
| 45 μg *Kcnt1* | 0.25 ± 0.04 (3) | 0.05 ± 0.01 (3) |
| 60 μg *Kcnt1* | 0.26 ± 0.06 (5) | 0.06 ± 0.03 (4) |
| 75 μg *Kcnt1* | 0.27 ± 0.09 (4) | 0.05 ± 0.01 (3) |
